# Supplementary material for: Intense training prevents the amnestic effect of inactivation of dorsomedial striatum and induces high resistance to extinction
Source: PLoS One. 2024 Jun 6;19(6):e0305066. doi: 10.1371/journal.pone.0305066 (PMC11156383; doi:10.1371/journal.pone.0305066)
Supplement: S1 Raw data — (PDF) [file pone.0305066.s001.pdf]

Infusion of TTX into dorsomedial striatum disrupts memory consolidation

Figure 2:

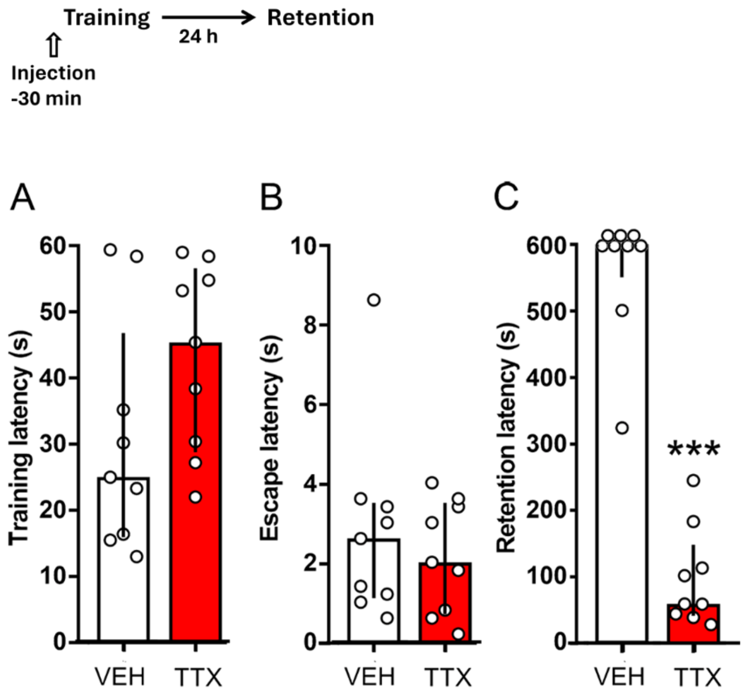

Table 1. Latency data in seconds of the effect of pre-training infusions of TTX into the dorsomedial striatum on memory consolidation of one-trial inhibitory avoidance trained with 1.0 mA.

	**Training**		**Escape**		**Retention**	
	VEH	TTX	VEH	TTX	VEH	TTX
	30	58.8	2.6	4	600	43.8
	58.2	53	3.6	0.8	600	245
	35	38.2	1	1.8	600	58.8
	12.8	21.8	3	2	600	58.6
	59.2	58.2	3.4	0.2	501.2	183.2
	16.2	45.2	8.6	3	600	113
	24.8	27	0.6	0.6	324.4	27.8
	23.1	54.6	1.2	3.4	600	38.6
	15.3	30.2	1.4	3.6	600	101.6
**Median**	**24.8**	**45.2**	**2.6**	**2**	**600**	**58.8**

TTX did not induce state-dependency

Figure 3:

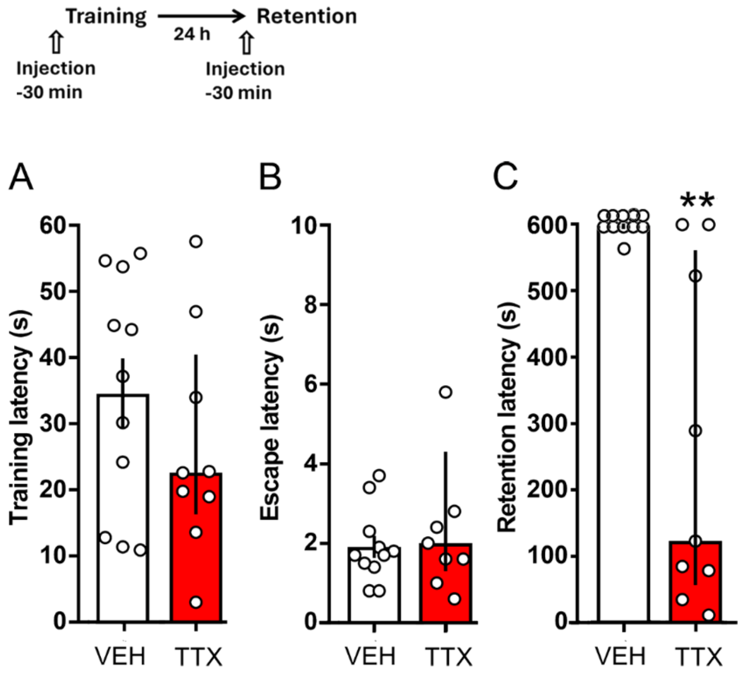

Table 2. Latency data in seconds of the test for state-dependency

	**Training**		**Escape**		**Retention**	
	VEH	TTX	VEH	TTX	VEH	TTX
	30.2	13.6	0.8	1	600	289.2
	11.4	19	3.4	2.4	600	84.4
	37.2	22.8	0.8	2.8	600	600
	10.9	22.6	3.7	2	563.6	600
	44.3	3	1.7	5.8	600	122.6
	44.9	57.6	2.3	0.6	600	34.4
	53.8	19.8	1.4	17	600	78
	54.7	34	1.8	1.6	600	10.8
	12.8	47	1.9	1.6	600	522.8
	55.8		1.5		600	
	24.2		1.7		600	
**Median**	**37.2**	**22.6**	**1.7**	**2**	**600**	**122.6**

TTX did not interfere with learning

Figure 4:

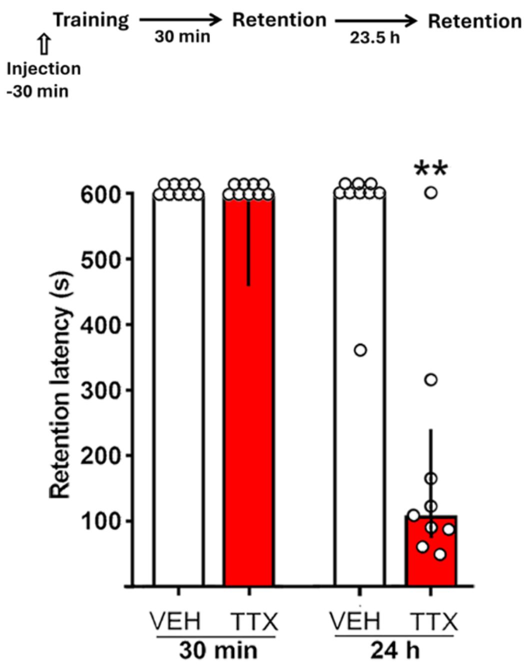

Table 3. Latency data in seconds of effect of the pretraining TTX administration into the dorsomedial striatum on short-term and long-term retention latencies

	**Retention 30 min**		**Retention 24 h**	
	VEH	TTX	VEH	TTX
	600	600	600	315
	600	266.2	600	87
	600	600	600	600
	600	316.4	600	60.7
	600	600	360	164.4
	600	600	600	108.4
	600	600	600	122.2
	600	600	600	48.9
	600	600	600	90
**Median**	**600**	**600**	**600**	**108.4**

Intense training protects against the amnestic effect of TTX

Figure 5:

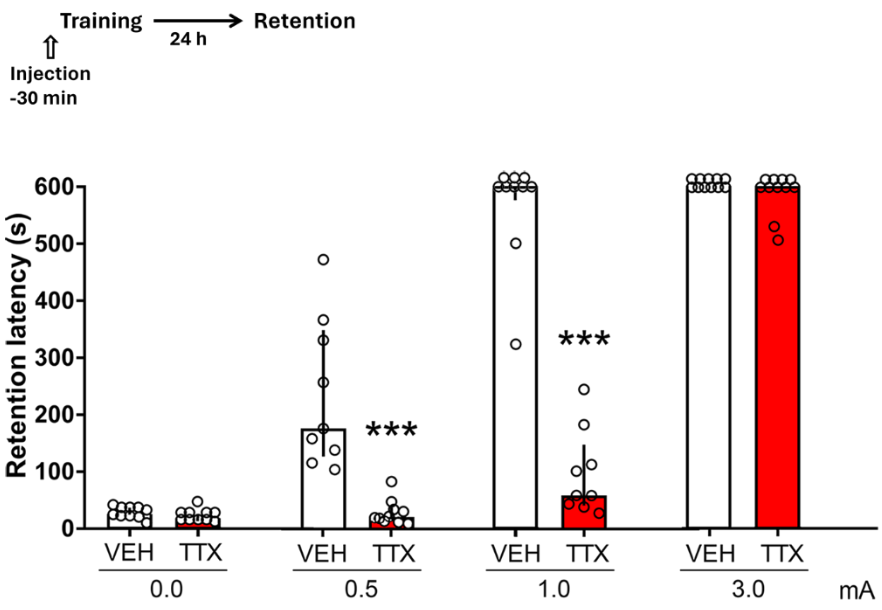

Table 4. Latency data in seconds of effects of pre-training infusions of TTX into the dorsomedial striatum on retention during the first extinction session of one-trial inhibitory avoidance trained with no foot shock (0.0 mA), or with low (0.5 mA), moderate (1.0 mA), and high (3.0 mA) foot shock intensities.

	**0.0 mA**		**0.5 mA**		**1.0 mA**		**3.0 mA**	
	VEH	TTX	VEH	TTX	VEH	TTX	VEH	TTX
	31.5	18.2	159.2	22.6	600	43.8	600	600
	7	16.4	177	9.8	600	245	600	600
	35	9.8	331.8	20.4	600	58.8	600	600
	18	46.6	258	12.4	600	58.6	600	600
	24.2	16.6	367.6	83.8	501.2	183.2	600	505.6
	21	24.2	139	31.2	600	113	600	529
	37.8	24.6	105	48.4	324.4	27.8	600	600
	20.4	21.4	116.4	34.8	600	38.6	600	600
	24.6	14.6	473.4	19.4	600	101.6	600	600
	38.8	14.6		14.3	600		600	600
							600	600
**Median**	**24.4**	**17.4**	**177**	**21.5**	**600**	**58.8**	**600**	**600**

Intense training produces high resistance to extinction after intra-DMS TTX administration

Figure 6:

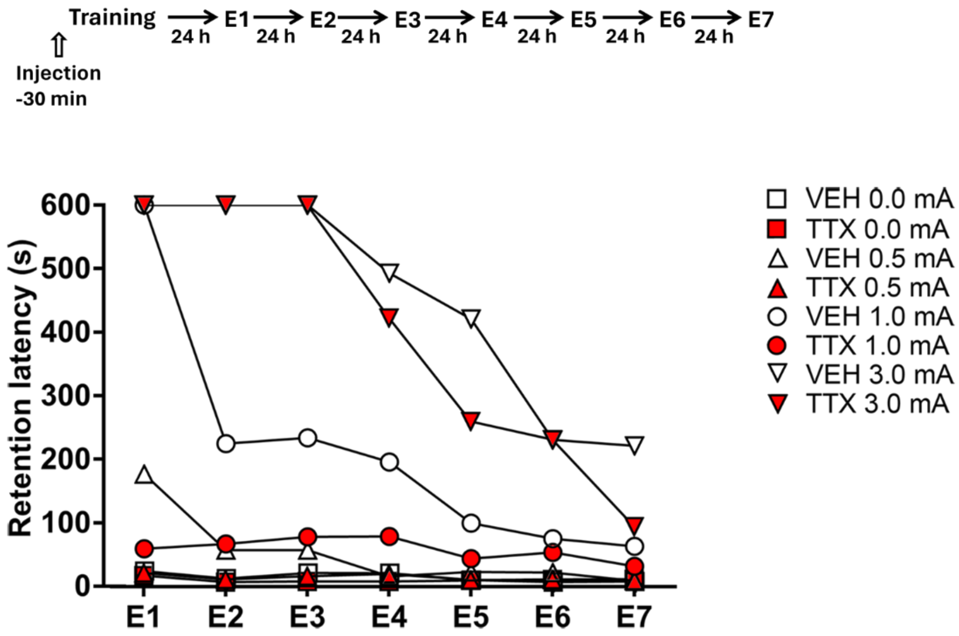

0.0 mA

Table 4. Latency data in seconds of effects of median retention latencies across the daily extinction sessions (E1–E7) shown by the groups trained in one-trial inhibitory avoidance with 0.0 mA.

	**E1**		**E2**		**E3**		**E4**		**E5**		**E6**		**E7**	
	VEH	TTX	VEH	TTX	VEH	TTX	VEH	TTX	VEH	TTX	VEH	TTX	VEH	TTX
	31.5	18.2	12.4	4	16.6	37.6	25.3	20.4	10.2	22.8	6.2	15	8.8	18.6
	7	16.4	4	12.3	59.8	21.8	24.4	12.2	8.2	20.2	5.6	5.6	10.6	2.8
	35	9.8	4.1	3.8	13.6	4.6	20.4	2.2	27.6	7	8.2	13.4	5	3
	18	46.6	18	23.6	71.8	4.4	50.4	7.2	27.1	12.8	15.4	10	5.4	21.2
	24.2	16.6	12.4	7.8	14	15	3.4	4.2	10.6	7.1	9.4	6.2	3.6	6
	21	24.2	24.6	7.4	73	2.8	15.7	4.4	58.8	11	200.8	9.4	181.8	14.2
	37.8	24.6	7.6	9.4	26.2	29.6	21.8	30.2	3.4	17.6	2.2	5.4	7.6	9.2
	20.4	21.4	20.8	7	37	2.6	26.4	5.6	2.4	7.8	6.3	22.6	10.6	3.8
	24.6	14.6	11	5.6	13	2.4	5.6	44.2	10.8	3.4	2.4	12.6	8.6	15.8
	38.8	14.6	22.4	5.8	3.6	11.4	13.2	8.8	2.2	8.2	11.6	19.3	6	14.8
**Median**	**24.4**	**17.4**	**12.4**	**7.2**	**21.4**	**8**	**21.1**	**8**	**10.4**	**9.6**	**7.25**	**11.3**	**8.1**	**11.7**

0.5 mA

Table 5. Latency data in seconds of effects of median retention latencies across the daily extinction sessions (E1–E7) shown by the groups trained in one-trial inhibitory avoidance with 0.5 mA.

	**E1**		**E2**		**E3**		**E4**		**E5**		**E6**		**E7**	
	VEH	TTX	VEH	TTX	VEH	TTX	VEH	TTX	VEH	TTX	VEH	TTX	VEH	TTX
	159.2	22.6	57.2	6	69.2	7.2	197.4	50.2	216.9	22	201.2	10	375	15.1
	177	9.8	130	25.4	77	70.6	23.8	19.2	30.8	144.2	10.6	300.5	5.8	600
	331.8	20.4	211.6	64	125.4	67.6	68.6	168.8	36.2	40.2	27.4	64.5	34.2	13.8
	258	12.4	44.3	12.4	57.4	4.8	10.2	62.4	35.4	27.8	47.8	20.8	25	4.8
	367.6	83.8	79.6	10	68	22.4	54.4	11.8	8.8	11.2	15.8	12.2	16.6	4.4
	139	31.2	3.4	6.6	12	11.2	9.8	3.6	9	9.2	3.2	21.8	2.2	10.2
	105	48.4	50.3	16.6	7	16.6	9.2	5.4	20.6	8.8	4	2.2	9.2	28
	116.4	34.8	6.6	94.2	10.4	16	12.2	20.2	23	5.4	32.9	5	8	6.4
	473.4	19.4	153.4	8	14.5	64.8	16	22.4	4.6	7.8	22.1	6.8	3.4	8.4
		14.3		4.4		3.2		11.2		8.4		6.5		5.1
**Median**	**177**	**21.5**	**57.2**	**11.2**	**57.4**	**16.3**	**16**	**19.7**	**23**	**10.2**	**22.1**	**11.1**	**9.2**	**9.3**

1.0 mA

Table 6. Latency data in seconds of effects of median retention latencies across the daily extinction sessions (E1–E7) shown by the groups trained in one-trial inhibitory avoidance with 1.0 mA.

	**E1**		**E2**		**E3**		**E4**		**E5**		**E6**		**E7**	
	VEH	TTX	VEH	TTX	VEH	TTX	VEH	TTX	VEH	TTX	VEH	TTX	VEH	TTX
	600	43.8	69.8	600	231.8	15.4	113.8	126.4	40.4	40.6	34.2	53.2	17.8	8.6
	600	245	600	293	600	199.4	600	157.8	600	142.2	600	77.2	600	67.4
	600	58.8	150.2	94.4	49.8	168	185.2	48.4	105.2	22.4	21.6	6.2	30.2	9.8
	600	58.6	184	15.4	184.2	12.4	99.2	8.4	93.4	7.2	102	5	51.2	9.2
	501.2	183.2	198.8	92.2	165	77.8	37.6	95.6	21.2	43.4	47.6	56.7	22.4	68.8
	600	113	95.6	66.6	235.2	93	206	83.6	231.6	46.1	414	41.8	297	31.4
	324.4	27.8	250.2	8	426.2	34	296.6	7.5	14.8	9.6	19.4	22.3	15.8	7.4
	600	38.6	600	35.2	600	33	600	68.2	600	59	279.4	62.4	171.9	42.2
	600	101.6	600	51.4	101.1	156.2	40.1	78.2	43.2	123	43.4	87.6	74.2	77.2
	600		600		600		600		465.6		465.6		260.8	
**Median**	**600**	**58.8**	**224.5**	**66.6**	**233.5**	**77.8**	**195.6**	**78.2**	**99.3**	**43.4**	**74.8**	**53.2**	**62.7**	**31.4**

3.0 mA

Table 7. Latency data in seconds of effects of median retention latencies across the daily extinction sessions (E1–E7) shown by the groups trained in one-trial inhibitory avoidance with 3.0 mA.

	E1		E2		E3		E4		E5		E6		E7	
	VEH	TTX	VEH	TTX	VEH	TTX	VEH	TTX	VEH	TTX	VEH	TTX	VEH	TTX
	600	600	600	600	600	600	600	600	600	600	600	438.2	221	180.4
	600	600	600	600	600	425.4	600	422.2	600	268.6	600	544.2	493	152.1
	600	600	600	600	600	600	259.2	163.6	214.4	91.6	163.6	144	127.6	145.6
	600	600	600	600	452	600	198.4	600	114	600	230.4	275.6	193	93.8
	600	505.6	600	353.4	600	204	375	26.8	600	50.2	600	24.8	330.6	38.2
	600	529	600	600	600	569	600	553.8	600	221.2	600	146.2	600	49.4
	600	600	600	600	504.2	600	336.3	600	193.2	329.2	210.8	412.4	107	169.4
	600	600	600	600	600	600	492.8	262.6	149.6	259.2	137.8	230.6	52.2	35.8
	600	600	600	303.6	600	246.8	211	231	140.4	78.8	145.8	91.3	112.8	17.4
	600	600	600	600	600	309.4	523.6	247.8	600	81.6	149.2	62.7	331	37.4
	600	600	600	600	600	600	600	600	420.6	600	284.8	600	333	217.8
**Median**	**600**	**600**	**600**	**600**	**600**	**600**	**492.8**	**422.2**	**420.6**	**259.2**	**230.4**	**230.6**	**221**	**93.8**

Figure 7:

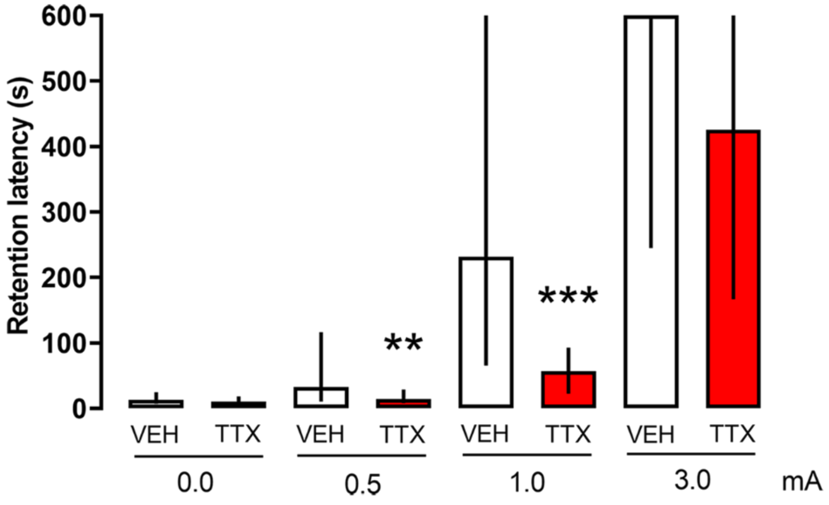

Table 8. Latency data in seconds of effect of pre-training infusions of TTX into the dorsomedial striatum on extinction of one-trial inhibitory avoidance trained with 0.0 mA, low (0.5 mA), moderate (1.0 mA), and high (3.0 mA) foot shock intensities.

	**0.0 mA**		**0.5 mA**		**1.0 mA**		**3.0 mA**	
	VEH	TTX	VEH	TTX	VEH	TTX	VEH	TTX
	31.5	18.2	159.2	22.6	600	43.8	600	600.0
	7	16.4	177	9.8	600	245.0	600	600.0
	35	9.8	331.8	20.4	600	58.8	600	600.0
	18	46.6	258	12.4	600	58.6	600	600.0
	24.2	16.6	367.6	83.8	501.2	183.2	600	505.6
	21	24.2	139	31.2	600	113.0	600	529.0
	37.8	24.6	105	48.4	324.4	27.8	600	600.0
	20.4	21.4	116.4	34.8	600	38.6	600	600.0
	24.6	14.6	473.4	19.4	600	101.6	600	600.0
	38.8	14.6	57.2	14.3	600	600.0	600	600.0
	12.4	4.0	130	6.0	69.8	293.0	600	600.0
	4	12.3	211.6	25.4	600	94.4	600	600.0
	4.1	3.8	44.3	64.0	150.2	15.4	600	600.0
	18	23.6	79.6	12.4	184	92.2	600	600.0
	12.4	7.8	3.4	10.0	198.8	66.6	600	600.0

	24.6	7.4	50.3	6.6	95.6	8.0	600	353.4
	7.6	9.4	6.6	16.6	250.2	35.2	600	600.0
	20.8	7.0	153.4	94.2	600	51.4	600	600.0
	11	5.6	69.2	8.0	600	15.4	600	600.0
	22.4	5.8	77	4.4	600	199.4	600	303.6
	16.6	37.6	125.4	7.2	231.8	168.0	600	600.0
	59.8	21.8	57.4	70.6	600	12.4	600	600.0
	13.6	4.6	68	67.6	49.8	77.8	600	600.0
	71.8	4.4	12	4.8	184.2	93.0	600	425.4
	14	15.0	7	22.4	165	34.0	600	600.0
	73	2.8	10.4	11.2	235.2	33.0	452	600.0
	26.2	29.6	14.5	16.6	426.2	156.2	600	204.0
	37	2.6	197.4	16.0	600	126.4	600	569.0
	13	2.4	23.8	64.8	101.1	157.8	504.2	600.0
	3.6	11.4	68.6	3.2	600	48.4	600	600.0
	25.3	20.4	10.2	50.2	113.8	8.4	600	246.8
	24.4	12.2	54.4	19.2	600	95.6	600	309.4
	20.4	2.2	9.8	168.8	185.2	83.6	600	600.0
	50.4	7.2	9.2	62.4	99.2	7.5	600	600.0
	3.4	4.2	12.2	11.8	37.6	68.2	600	422.2
	15.7	4.4	16	3.6	206	78.2	259.2	163.6
	21.8	30.2	216.9	5.4	296.6	40.6	198.4	600.0
	26.4	5.6	30.8	20.2	600	142.2	375	26.8
	5.6	44.2	36.2	22.4	40.1	22.4	600	553.8
	13.2	8.8	35.4	11.2	600	7.2	336.3	600.0
	10.2	22.8	8.8	22.0	40.4	43.4	492.8	262.6
	8.2	20.2	9	144.2	600	46.1	211	231.0
	27.6	7.0	20.6	40.2	105.2	9.6	523.6	247.8
	27.1	12.8	23	27.8	93.4	59.0	600	600.0
	10.6	7.1	4.6	11.2	21.2	123.0	600	600.0
	58.8	11.0	201.2	9.2	231.6	53.2	600	268.6
	3.4	17.6	10.6	8.8	14.8	77.2	214.4	91.6
	2.4	7.8	27.4	5.4	600	6.2	114	600.0
	10.8	3.4	47.8	7.8	43.2	5.0	600	50.2

	2.2	8.2	15.8	8.4	465.6	56.7	600	221.2
	6.2	15.0	3.2	10.0	34.2	41.8	193.2	329.2
	5.6	5.6	4	300.5	600	22.3	149.6	259.2
	8.2	13.4	32.9	64.5	21.6	62.4	140.4	78.8
	15.4	10.0	22.1	20.8	102	87.6	600	81.6
	9.4	6.2	375	12.2	47.6	8.6	420.6	600.0
	200.8	9.4	5.8	21.8	414	67.4	600	438.2
	2.2	5.4	34.2	2.2	19.4	9.8	600	544.2
	6.3	22.6	25	5.0	279.4	9.2	163.6	144.0
	2.4	12.6	16.6	6.8	43.4	68.8	230.4	275.6
	11.6	19.3	2.2	6.5	465.6	31.4	600	24.8
	8.8	18.6	9.2	15.1	17.8	7.4	600	146.2
	10.6	2.8	8	600.0	600	42.2	210.8	412.4
	5	3.0	3.4	13.8	30.2	77.2	137.8	230.6
	5.4	21.2		4.8	51.2		145.8	91.3
	3.6	6.0		4.4	22.4		149.2	62.7
	181.8	14.2		10.2	297		284.8	600.0
	7.6	9.2		28.0	15.8		221	180.4
	10.6	3.8		6.4	171.9		493	152.1
	8.6	15.8		8.4	74.2		127.6	145.6
	6	14.8		5.1	260.8		193	93.8
							330.6	38.2
							600	49.4
							107	169.4
							52.2	35.8
							112.8	17.4
							331	37.4
							333	217.8
**Median**	**13.1**	**10.5**	**32.9**	**14.05**	**231.7**	**56.7**	**600**	**425.4**
