# Supplementary material for: Intense training prevents the amnestic effect of inactivation of dorsomedial striatum and induces high resistance to extinction
Source: PLoS One. 2024 Jun 6;19(6):e0305066. doi: 10.1371/journal.pone.0305066 (PMC11156383; doi:10.1371/journal.pone.0305066)
Supplement: Attachment — Submitted filename: Plos one review.docx [file pone.0305066.s002.docx]

Dear Editor

Roberto et al. investigated the effect of intense training on memory and extinction. They reported that intense training induces resistance to the extinction.

I believed this manuscript is good but there are several concerned that should be attention by authors.

The main point is that this finding should be support by histological parameters. I think neurological index such as Neru-D.

Why did the researchers not use other learning tests such as the Morris Water Maze?

How did the researchers divide the intensity into low, medium and high? Is there a standard?

Point 1: PTSD abbreviation should be explaining previously.

Point 2: It is surprising that a 9-week-old animal has a 350 g weight.

Point 3: number of rats in each group and study design should be mention in materials and methods.

Point 4: The quality of figure 1 is not clear.
